# Patient and system factors of mortality after hip fracture: a scoping review

**DOI:** 10.1186/s12891-016-1018-7

**Published:** 2016-04-14

**Authors:** K. J. Sheehan, B. Sobolev, A. Chudyk, T. Stephens, P. Guy

**Affiliations:** School of Population and Public Health, University of British Columbia, Vancouver, Canada; Centre for Hip Health and Mobility, Vancouver, Canada; Department of Orthopaedics, University of British Columbia, Vancouver, Canada

**Keywords:** Scoping review, Hip fracture, Mortality, Patient factors, System factors

## Abstract

**Background:**

Several patient and health system factors were associated with the risk of death among patients with hip fracture. However, without knowledge of underlying mechanisms interventions to improve survival post hip fracture can only be designed on the basis of the found statistical associations.

**Methods:**

We used the framework developed by Arksey and O’Malley and Levac et al. for synthesis of factors and mechanisms of mortality post low energy hip fracture in adults over the age of 50 years, published in English, between September 1, 2009 and October 1, 2014 and indexed in MEDLINE. Proposed mechanisms for reported associations were extracted from the discussion sections.

**Results:**

We synthesized the evidence from 56 articles that reported on 35 patient and 9 system factors of mortality post hip fracture. For 21 factors we found proposed biological mechanisms for their association with mortality which included complications, comorbidity, cardiorespiratory function, immune function, bone remodeling and glycemic control.

**Conclusions:**

The majority of patient and system factors of mortality post hip fracture were reported by only one or two articles and with no proposed mechanisms for their effects on mortality. Where reported, underlying mechanisms are often based on a single article and should be confirmed with further study. Therefore, one cannot be certain whether intervening on such factors may produce expected results.

## What is previously known

- Excess mortality persists for years post hip fracture.
- Several patient and system factors have been associated with the risk of death in patients with hip fracture.
- However underlying mechanisms of the found associations are rarely discussed.

## What this study adds

- We synthesized the evidence from 56 recent articles that reported on 35 patient and 9 system factors of mortality post hip fracture.
- The majority of factors were reported with no proposed mechanisms for their effects on mortality. Where reported, underlying mechanisms are often based on a single article.
- The proposed biological mechanisms include complications, comorbidity, cardiorespiratory function, immune function, bone remodeling and glycemic control.

## Background

Hip fracture is a leading cause of injury related mortality in older adults [1]. Omsland and colleagues reported mortality rates five times higher in men and three times higher in women compared to the general population in the first year post fracture [2]. This excess mortality persists 10 years post fracture [2, 3].

The existing literature identifies patient and system factors associated with the risk of death among patients with hip fracture. However, no attempt has been made to synthesize this literature on the underlying mechanisms for these associations.

Without knowledge of mechanisms mediating an association, interventions to improve survival post hip fracture can only be designed on the basis of the reported statistical associations. We believe designing interventions should rely on knowledge about a modifiable factor with negative effect on survival. Where modifiable factors lie on the causal pathway between exposure and outcome determines the focus of an intervention.

Scoping reviews represent an approach to summarizing the range of evidence on a subject, to clarify a complex concept, and to help refine subsequent research questions for a full systematic review [4, 5]. This review contributes to the existing literature by synthesizing the evidence available on patient and system factors of mortality after hip fracture. To go beyond traditional reports, we extract and synthesize additional information on biological and hypothetical mechanisms for reported associations. More specifically, the aims of this review are 1) to identify patient and system factors of mortality after hip fracture, and 2) collate the description of proposed mechanisms for their associations with mortality.

## Methods

The key elements of the scoping review framework include formulating the research question, identifying relevant studies, selecting studies from electronic database, charting the extracted data and collating, summarizing and reporting findings. We extend this framework by collecting information on the underlying mechanisms for found associations. We have followed to a widely recognized framework by Arksey and O’Malley [6] and recommendations of Levac et al. [5] for conducting and reporting scoping reviews. This scoping review synthesizes published literature and ethical approval was not required.

The population of interest is frail adults aged 50 years or older admitted to acute care with non-pathologic low energy hip fracture. Concepts of interest include both patient and system factors. The outcome of interest is mortality following usual care.

### Study selection

One reviewer searched MEDLINE using the search terms “mortality” Medical Subject Headings [MeSH] AND “hip fracture” [MeSH] and screened studies for eligibility. Studies were first screened according to title and abstract with those that appeared suitable selected for a full-text review using standardized inclusion criteria (Table 1). Studies marked as ‘maybe for inclusion’ were screened by a second reviewer for eligibility.

**Table 1:** Inclusion criteria for the literature search

Term	Include
Study population	Men & women ≥50 years of age with non-pathological low energy hip fracture
Study design	0bservational studies
Factors	Patient and system factors of mortality
Associations	Estimates from regression analysis
Outcome	Mortality (in-hospital, 30 day, 12 month, >12 month)
Date	Between Sep 1, 2009 and Oct 1, 2014
Language	English
Geography	Worldwide

We included reports from 2009 or later to minimize the potential biasing effects of demographic aging [7–9], surgical advancements [10], and changes in delivery of hip fracture care [11–13]. Intervention-based studies were excluded as they do not reflect hip fracture mortality following usual care. Studies whose main independent variables were laboratory tests or operation type were also excluded as they were considered outside of the current scope of interest. Finally, only studies which conducted a regression analysis were included as regression analysis was deemed a proxy for adequate sample size [14].

Using a formal instrument, one reviewer extracted authors name, publication date, timing of assessment relative to the hip fracture event, length of follow up, patient and system factors from each article. The significance of statistical associations between the factors and mortality was derived from the 95 % confidence intervals reported in the articles. The proposed mechanisms for mortality were extracted from discussions by one reviewer. The accuracy of extraction was assessed by a second reviewer.

### Collating, summarizing and reporting results

Patient and system factors of mortality studied in the reviewed articles are summarized in Tables 2, 3 and 4. Factors with a proposed mechanism of their effects on mortality are summarized in Tables 5 and 6 with indication whether the mechanisms is hypothetical.

**Table 2:** Articles studying mortality in relation to injury and complications

	Fracture type	Injury severity	Additional trauma	Shock	Complications	Cardiovascular complications	Decubitus ulcer	Gastrointestinal bleeding	Pulmonary complications	Clostridium difficile	Renal failure	Pneumonia	Delirium
Belmont 2014 [15]		√		√									
Neuhaus 2013 [17]	√		√										
Miller 2012 [22]						√							
Gold 2012 [24]							√						
Librero 2012 [23]		√											
Tarazona-Santabalbina 2012 [47]													√*
Lee 2011 [62]													√
Miyanishi 2010 [63]					√								
Vaseenon 2010 [65]	√*												
Juliebo 2010 [66]													
Rahme 2010 [55]								√			√		
Lapcevic 2010 [57]									√		√		
Juliebo 2010 [66]													√*
Berry 2009 [60]							√					√	
Gulihar 2009 [41]										√			
Among all	1	2	1	1	1	1	2	1	1	1	2	1	1

*no statistical association found

**Table 3:** Articles studying mortality in relation to demographic factors and comorbidity

	Age	Sex	Race	Preadmission residence	Functional status	Any comorbidity	Liver disease	Diabetes	Malignancy	Malnutrition	Low Body Mass Index*	Obesity	SecondaryHyperparathyroidism**	Cardiac disease	Cardiac arrhythmia	Congestive heart failure***	Coronary artery disease^¥^	Myocardial infarction^§^	Cerebrovascular accident^¶^	Anemia	Cognitive impairment	Dementia
Belmont 2014 [15]		√												√								
Neuhaus 2013 [17]	√	√				√									√							
Williams 2013 [36]	√	√				√																√
Hagino 2013 [16]										√												
Talsnes 2013 [37]						√																
Uzoigwe 2013 [19]	√	√		√		√																
Clement 2013 [42]						√																
Daugaard 2012 [18]	√	√				√																
Le-Wendling 2012 [20]	√	√	√																			
Librero 2012 [23]	√	√				√																
Huddleston 2012 [44]		√														√		√				√
Adunsky 2012 [43]	√	√													√							
Gupta 2012 [45]																		√				
Valizadeh 2012 [46]	√	√****			√	√****																
Tarazona-Santabalbina 2012 [47]	√	√			√	√															√	
Pioli 2012 [48]					√																	
Sanz-Reig 2012 [49]					√	√							√									√
Vidan 2011 [25]	√				√	√																√
Koval 2011 [26]		√				√																
Frost 2011 [27]	√	√				√	√									√						
Kirkland 2011 [38]						√																
Carretta 2011 [39]	√	√				√					√				√				√			
Gulcelik 2011 [52]								√														
Talsnes 2011 [53]	√	√				√																
Baker 2011 [64]																						√
LeBlanc 2011 [70]	√																					
Holvik 2010 [54]				√		√																
Kesmezacar 2010 [67]	√																					
Rahme 2010 [55]	√	√		√		√		√	√						√	√	√					
Forte 2010 [56]	√		√			√																
Lapcevic 2010 [57]	√	√					√		√	√						√						√
Miyanishi 2010 [63]											√											√
Juliebo 2010 [66]		√			√						√				√		√					
Jamal 2010 [59]		√										√										
Bjorgul 2010 [69]	√	√				√																
Pereira 2010 [58]	√	√			√	√****		√											√			
Vaseenon 2010 [65]		√																				
Berry 2009 [60]	√	√			√											√	√			√		
Lefaivre 2009 [33]	√	√				√																
Vidal 2009 [35]	√	√				√																
Feng 2009 [68]						√																
Among all	23	23	2	3	8	23	2	3	2	2	3	1	1	1	5	5	3	2	2	1	1	7

*Body mass index

** Secondary hyperparathyroidism

*** Congestive heart failure

****no statistical association found

^¥^ Coronary artery disease

^§^ Myocardial infarction

^¶^ Cerebrovascular accident

**Table 4:** Articles studying mortality in relation to system factors

	Hospital volume	Surgeon volume	Nursing volume	July admit	General anesthetic	Intensive care admit	Short stay	Hospitalization delay	Surgical delay
Belmont 2014 [15]									√*
Li 2014 [61]								√	√
Uzoigwe 2013 [19]									√
Williams 2013 [36]							√		√
Neuman 2012 [21]					√				
Pioli 2012 [48]									√
Vidal 2012 [50]								√	√*
Tarazona-Santabalbina 2012 [47]				√*					√*
Le-Wendling 2012 [20]						√			
Sanz-Reig 2012 [49]									√*
Daugaard 2012 [18]									√
Koval 2011 [26]									√
Peleg 2011 [30]									√
Schilling 2011 [28]			√						
Carretta 2011 [39]									√
Forte 2010 [56]	√	√							
Kesmezacar 2010 [67]									√
Browne 2009 [34]	√*	√							
Anderson 2009 [31]				√					
Vidal 2009 [35]					√				
Among all	1	2	1	1	2	1	1	2	9

*no statistical association found

**Table 5:** Proposed mechanisms and mediators for the mortality effect of patient factors

Factor	Mechanism	Mediator
Age	Aging reduces the reserve capacity necessary to cope with a double trauma of hip fracture and surgery [22, 37].	Hypothesis only
	The number of chronic conditions increases with age [47, 70].	Extent of comorbidity
Sex	Men present with more comorbidity than women [47, 54, 65, 68].	Extent of comorbidity
	Men develop delirium [60], lung infection, pneumonia, and septicemia more often than women [54, 68].	Complications
Prefracture function	Patients with poorer pre-fracture ambulatory status often have reduced cardiorespiratory function compared to those with better status [68].	Cardiorespiratory function
	Patients with a high degree of dependency are more often delayed to admission than patients with a low degree of dependency [50].	Hospitalization delay
	Patients with poor pre-fracture ambulatory status are quickly placed in nursing homes while patients with better status wait in hospital for rehabilitation beds [36].	Length of stay
Preadmission residence	Institutionalized patients develop pneumonia and pressure ulcer more often than patients from community [54, 60].	Complications
Socioeconomic status	Patients with low socioeconomic status are more often delayed to admission than patients with high socioeconomic status [50].	Hospitalization delay
Clinical stability	Patients who are acutely unstable on admission are delayed to surgery more often than those who are stable [25, 39].	Surgical delay
Extent of comorbidity	Multiple comorbidities diminish reserves for stresses of surgery and delays recovery [37, 38].	Hypothesis only
	Patients with more comorbidity are delayed to surgery more often than those with less comorbidity [25, 48].	Surgical delay
	Patients with more comorbidity are quickly placed in nursing homes while patients with less comorbidity wait in hospital for rehabilitation beds [36].	Length of stay
Body composition	Patients with low BMI are more likely to develop adverse cardiac event post hip fracture surgery [66].	Complications
	Patients with low BMI are more likely to be frail [66] and have diminished reserves to cope with the stress of surgery [38].	Hypothesis only
	Patients with low BMI often have reduced cardiorespiratory function and a supressed immune system [38].	Immune response, Cardiorespiratory function
History of cerebrovascular accident	Patients with hemiplegia often have more comorbidity and poor pre-fracture ambulatory status [68].	Extent of comorbidity, Pre-fracture function
Dementia	Patients with dementia often have more comorbidity and poor pre-fracture ambulatory status [68].	Extent of comorbidity, Pre-fracture function
Diabetes	Diabetes may lead to poor bone remodeling post hip fracture [52].	Bone remodeling [77]
	Diabetes may lead to poor wound healing post hip fracture surgery [52].	Hypothesis only
	Patients with diabetes may have poor glycemic control leaving the body prone to infections and complications after surgery [52].	Glycemic control [78] Complications
Malnutrition	Patients with malnutrition often present with more comorbidity and poor pre-fracture ambulatory status.(16;38)	Extent of comorbidity, Pre-fracture function
Myocardial infarction	Patent foramen ovale allows procoagulant cell conjugates and fragments to pass directly from the venous to the arterial blood [37].	Hypothesis only
Secondary hyperparathyroidism	Patients with secondary hyperparathyroidism often have more comorbidity [51].	Extent of comorbidity
	Secondary hyperparathyroidism leads to severely altered calcium homeostasis [32].	Calcium homeostasis

**Table 6:** Proposed mechanisms and mediators for the effect of system factors on mortality

Factor	Mechanism	Mediator
Hospital volume	Patients admitted to low volume hospitals are often delayed to surgery when compared to patients admitted to high volume hospitals [56].	Surgical delay
Nursing staff volume	Higher nurse staffing may prevent or allow early detection of complications [28].	Complications
	Higher nurse staffing improves operating room availability and shorten time to surgery [28].	Surgical delay
Surgeon volume	Low volume surgeons may not select appropriate procedure and preoperative planning, intraoperative technique and postoperative management [34].	Hypothesis only
Surgical delay	Patients who are delayed to surgery are exposed to inflammatory and hypercoagulable states for longer than those who are not delayed [71, 72].	Hypothesis only
Hospitalization delay	Patients may receive suboptimal care prior to admission and may develop pressure ulcers, thromboembolism, uncontrolled pain or delirium [50, 61].	Complications
Length of stay	Institutionalized patients have shorter hospital stay than patients from community [36].	Discharge destination
Admission month	Patients admitted in July may be exposed to lower staffing levels in holiday period [31].	Staffing volume

## Results

### Search results

**Fig. 1:**
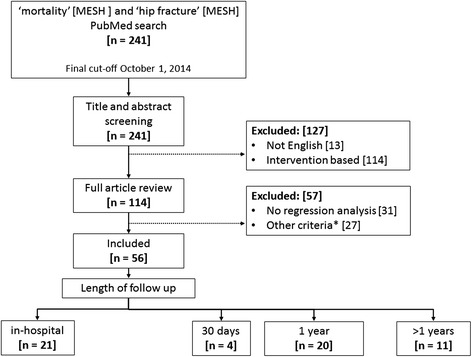
Flow chart of the literature retrieval, review, exclusion and selection with sorting by follow up time. n = number. * = Studies excluded with patient populations less than 50 years old, pathological or high impact hip fractures, or whose main independent variables were laboratory tests or operation type

The search produced 241 articles for initial title and abstract screening. Figure 1 shows the selection process which identified 56 articles used in this review. Among the selected articles, 21 reported on in-hospital mortality [15–35], 4 reported on 30 day mortality [36–39], 20 reported on 12 months mortality [40–59], and 11 reported more than 12 month mortality [60–70].

### Patient factors of mortality

We identified 35 patient factors of mortality post hip fracture reported in the reviewed articles, Tables 2 and 3. The majority of factors were studied by only one or two studies included in this review. There is a general consensus in the literature that mortality is associated with age, sex, comorbidity, functional status, dementia, arrhythmia and congestive heart failure. We noted conflicting reports for the association between mortality and both fracture type [17, 65] and delirium [47, 62, 66].

**Fig. 2:**
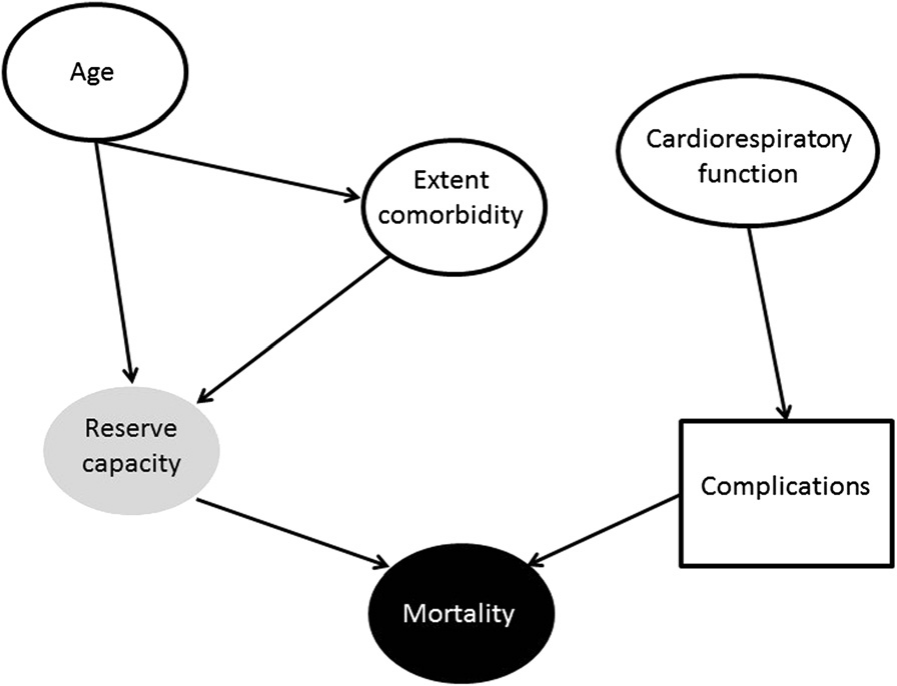
Examples of mechanisms proposed for patient factors in reviewed articles. Black node indicates the outcome. White nodes indicates a reported factor. Square box indicates a measurable mediator. Grey node indicates a hypothetical mediator

For 14 factors we found a proposed mechanism of their effects on mortality, Table 5. Biological mechanisms included comorbidity [47, 70], cardiorespiratory function [68], immune function [38], bone remodeling [52], glycemic control [52], and calcium homeostasis [32]. Non-biological mechanisms included hospitalization delay [50], surgical delay [25, 48] and length of stay [36]. Some proposed included hypothetical mediators, such as reduced reserve capacity [22, 37], a patent foramen ovale [37] and reduced wound healing [52]. Fig. 2 shows two examples of the mechanisms proposed for patient factors in the reviewed articles. First, the onset of complications mediates the effect of cardiorespiratory function on mortality [63]. Second, a hypothetical reduction in reserve capacity mediates the mortality effect of age and extent of comorbidity [22, 37].

### System factors of mortality

In the reviewed articles, we identified 9 system factors of mortality post hip fracture including hospitalization delay, July admission, surgical delay, anaesthetic type, intensive care admission, hospital volume, surgeon volume, nursing volume and length of stay, Table 4. There is no consensus in the literature on system factors of mortality. The most studied factor was surgical delay (9 articles). However, the association of mortality with surgical delays is disputed by reports of no association [15, 47, 49, 50]. We also noted conflicting reports for the association between mortality and both July admission and hospital volume. The other factors were studied by only one or two studies included in this review.

For 7 factors we found a proposed mechanism of their effects on mortality, Table 6. Complications were proposed as a biological mechanism for the mortality effect of nursing staff volume [28] and hospitalization delay [50, 61]. Non-biological mechanisms included surgical delay [28], staffing volume [31] and discharge destination [36]. Some proposed included hypothetical mediators, such as, exposure to inflammatory and hypercoagulable states [71, 72] and inappropriate planning, technique or management [34].

## Discussion

The purpose of this review was to synthesize the information available on proposed mechanisms for reported associations between patient and system factors and mortality after hip fracture. The articles included in this review point to plausible mediators in the biological mechanisms for mortality post fracture: complications, comorbidity, cardiorespiratory function, immune function, bone remodeling and glycemic control. For example, exposure to immobilization and inflammatory states is the proposed mechanism mediating the mortality effect of hospitalization delay [71, 72]. As argued elsewhere, prolonged immobilization leads to potentially fatal complications such as pulmonary embolism and pneumonia while prolonged hypercoagulable inflammation leads to potentially fatal complications including stroke and myocardial infarction [73].

A hypothetical reduction in reserve capacity, whereby a patient cannot withstand the stress of trauma and their pre-existing comorbidity [38], was proposed as a mechanism for the mortality effect of comorbidity [22, 37]. It seems plausible, because numerous studies associated mortality with coexisting arrhythmia, congestive heart failure, coronary artery disease, myocardial infarction, anemia and cerebrovascular accident. As noted elsewhere, patients undergoing hip fracture surgery require the reserve capacity to withstand the cardiovascular depressant effect of anaesthesia [74]. For those who survive beyond the short-term, patients with cardiovascular disease more often present with reduced reserve exercise capacity [75] compromising their rehabilitation potential and placing them at greater risk of dependency, complications and death [76].

This is the first scoping review to synthesize the proposed biological and hypothetical mechanisms for patient and system factors of mortality following hip fracture. Such synthesis represents a first step towards transparency about underlying assumptions when informing policy on potential interventions to improve survival in this vulnerable population.

This review is not without limitations. In contrast to a systematic review, where literature is critically appraised on the methodology, we assess the reviewed articles only according to the presence of proposed mechanisms for the reported associations. This is a common approach in scoping reviews where the purpose is to collate the evidence on a topic of interest. [5] The search strategy was restricted to one database over a 5 year period preceding the review development to minimize the potential biasing effects of surgical advancements [10], and changes in delivery of hip fracture care [11–13]. We excluded articles reporting outcomes of interventions as they do not reflect hip fracture mortality resulting from usual care. These restrictions may result in lacking some articles both on factors of mortality and proposed mechanisms.

## Conclusions

We synthesized proposed mechanisms for reported associations between patient and system factors and mortality after hip fracture. We identified complications, comorbidity, cardiorespiratory function, immune function and bone remodeling and glycemic control as plausible mediators in the biological mechanisms for mortality post fracture. However, we found that the majority of patient and system factors of mortality post hip fracture were reported by only one or two articles and with no proposed mechanisms for their effects on mortality. Where reported, underlying mechanisms are often based on a single article and should be confirmed with further study. Therefore, one cannot be certain whether intervening on such factors may produce expected results.

### Ethical approval and consent to participate

Not applicable.

### Consent for publication

Not applicable.

### Availability of data and materials

Articles were identified in MEDLINE. All articles included in the final review are identified in the reference list. Access to full text for each article is dependent on journal and institutional constraints.

## Abbreviation

MESH: medical subject headings
